# Retraction: Unleashing the role of e-word of mouth on purchase intention in select Facebook fan pages of smart phone users

**DOI:** 10.1371/journal.pone.0331054

**Published:** 2025-11-05

**Authors:** The PLOS One Editors

Following the publication of this article [1], similarities were noted between this article and a previously published thesis [2]. During editorial follow up the first author stated that the article [1] replicated an existing work and does not make a new contribution to the literature; they requested retraction on the basis of plagiarism.

In light of these issues, the *PLOS One* Editors retract this article [1].

All authors agreed with the retraction.

The retracted article was removed from the *PLOS One* website at time of retraction. The article’s Copyright and Data Availability statements were updated at the time of retraction and removal, and the removed contents are no longer offered under the Creative Commons Attribution License. Readers should refer to [2] for the copyright notice.
