# Correction: Retraction: Unleashing the role of e-word of mouth on purchase intention in select Facebook fan pages of smart phone users

**DOI:** 10.1371/journal.pone.0350593

**Published:** 2026-06-01

**Authors:** The PLOS One Editors

The *PLOS One* Editors issue this notice to correct an error in the retraction notice [1] for this article [2]. Contrary to the statement in the retraction notice, the article was not removed from the *PLOS One* website at the time of retraction, but instead was removed on May 18, 2026. The article’s [2] Copyright and Data Availability statements have now been updated to note the removal.

The publisher apologizes for the error.
